# Differential Leucocyte Counts Which May Assist Diagnosis

**Published:** 1907-09-28

**Authors:** 


### Points in Diagnosis.

### DIFFERENTIAL LEUCOCYTE COUNTS WHICH MAY ASSIST DIAGNOSIS.

(*Continued from page* 450.)

Malaria is diagnosed with absolute certainty only when the hæmatozoa themselves are seen in the blood. When very numerous they can be found in fresh blood without staining; more often it is necessary to make a blood-film by one of the methods described in the article on Eosinophilia on page 9 of our issue of April 6, and to stain it. Jenner's stain may be used, as described in the same article, but a still better stain for malaria parasites is Leishman's. The formula for Leishman's stain is as follows:—

| | |
|---|---|
| Crystalline methylene-blue-eosin compound (Leishman's modification) ... | 0.2 grams |
| Methyl alcohol ... ... ... ... | 100 c.c. |

The stain may be obtained from any large firm of manufacturing chemists; it is used in the following way:—

The microscope slide is laid upon a horizontal surface with the film-side upwards. It is very important to have a cover, such as a Petrie dish, which can be put over the slide during the process to prevent evaporation and consequent deposition of solid particles of stain. A small funnel with a suitable filter paper is required, and some Leishman's stain is poured into the filter, and the freshly filtered fluid allowed to drop on to the film until the latter is completely and evenely covered by it. Then, after standing for one minute, pure distilled water is allowed to drop into the stain over the film, drop by drop, until distilled water has been added equal to the volume of the stain originally placed on the slide. By an oscillating movement the stain and the distilled water are now evenly mixed, and the slide is allowed to stand for fully another five minutes, and it does not matter if it is left standing even a minute or two longer. The diluted stain is then rapidly poured off the slide, and the latter is quickly washed with clean distilled water; the slide should be left lying horizontal with some distilled water upon it for one minute. It should then be held vertically with its lower end in contact with blotting paper; this will remove all the water that will drain off spontaneously, the drying of the film being completed by allowing the slide to stand in a current of air. It should not be warmed or heated. When dry, the film can be covered with Canada balsam and a cover-glass in the usual way; or it can be examined without being covered, cedar-wood oil being put directly on to the stained film.

It is essential that a $\frac{1}{12}$th-inch oil-immersion lens should be used in searching the films for the parasites, and a mechanical stage is a very great advantage. The parasites, if they are present, will be easily recognised either as pale-blue bodies containing dark-brown or almost black pigment-granules within the red corpuscles, or as crescentic bodies containing similar pigment granules, the crescents either lying free, or each having attached to its concave side the "ghost" of a red corpuscle. There are excellent pictures of these parasites in most text-books of medicine, and they need not be repeated here. It will depend upon the phase of the malaria what size and form the parasites will present; they are at their maximum development just before the initial rigor of an ague fit, and during the fit itself they are at their minimum of development, sporulation having just occurred, so that there may be no parasites within the blood corpuscles at all.

It may be worth while to give, briefly, the life-history of the hæmatozoon. There are two cycles—a sexual and an asexual. The sexual cycle occurs within the mosquito; the asexual in man. The stages of each cycle are as follows:—

1. The infected mosquito bites a man, and through its proboscis introduces fine spores, termed *ex-oto-spores*, into the man's blood. Each such ex-oto-spore is a potential malaria parasite; it becomes amæboid, invades a red blood corpuscle, appearing at first as a pale excentric speck, but presently growing at the expense of the protoplasm of the red blood disc until it becomes—

2. A typical *hæmatozoon*, nearly filling the red corpuscle in which it is, staining blue with Leishman's stain, and containing near its centre a group of dark-brown pigment granules. When fully grown the protoplasm of the hæmatozoon divides into segments arranged radially from the centre, where the pigment granules are; this form of the parasite is termed—

3. The *rosette*; just when the rigor of an ague fit comes on, the rosette bursts asunder, setting free its segments or spores as—

4. *En-hœmo-spores*; except that they are formed in the blood of the patient instead of in the body of the mosquito these are identical with the ex-oto-spores; and, like the ex-oto-spore, each enhœmo-spore again becomes amœboid, invades a red corpuscle, grows, produces pigment granules, divides into a rosette at the time of the next rigor, and liberates a fresh series of en-hœmo-spores; and so on, and so on, each such asexual cycle corresponding to one ague fit. There are some minor microscopical differences between the tertian and the quartan parasite, but except in the duration of the cycle, the life-history of the two is very similar. The rosette of the quartan parasite contains fewer spores and larger and fewer pigment granules than does the corresponding rosette of the tertian.

When a mosquito bites the infected patient, and takes into its stomach some of the asexual parasites, there ensues in the mosquito—

1. The first stage of the sexual cycle may sometimes be observed in fresh malarial blood that has been allowed to stand for an hour without drying. Some of the hæmatozoa throw out long thin flagellum-like processes; these are motile, and are the *male* elements, corresponding exactly to the spermatozoa of mammals. Other hæmatozoa extrude from themselves two polar bodies, and become the *female* elements, corresponding to the ovum of mammals.

2. In the mosquito stomach the flagellum-like male element enters the female element from which the polar bodies have been extruded, the result being a fusion of the male and female elements—that is to say, a fertilised ovum.

3. The fertilised ovum becomes elongated and motile, and from its appearance is termed the *vermicule* or little worm; this bores its way through the stomach wall of the mosquito, and comes to lie in the sinuses outside the stomach. It here loses motility, and begins to grow and subdivide, forming a *sporocyst* and containing a mass of small cells, each of which is called a—

4. *Spore-mother-cell*; in due course each spore-mother-cell breaks up into a number of *spores*. These become liberated within the sinuses around the stomach. By the circulating fluids some of the spores are carried into the proboscis of the mosquito, and when the infected mosquito bites a man some of them become transferred to the man as *exoto-spores*, similar to those with which we started; the asexual cycle is then ready to begin again.

Conclusion.

In all cases of suspected malaria the first thing to do in order to confirm the diagnosis, is to examine the blood for the parasites; and since these are at their fullest development just before the rigor, this is the best time to make the blood film. In the tropics many parasites will almost certainly be present, and they will in all probability be readily found. The chief difficulties arise when the patient has returned to this country, having formerly had malaria abroad, and, after a longer or shorter interval, has a rigor, which might be a recurrence of the malaria, or, alternatively, might be something else. In such cases the detection of the parasites in a film may be either difficult or impossible for at least three reasons:—

1. The total number of parasites in the blood may be so few that probably none will be present in a single film of blood, or even in two.

2. The rigors may be very irregular, and the films may thus be taken at a time when the parasites are undeveloped, and therefore difficult to recognise.

3. The patient may have taken quinine, or, owing to the recurrence of the rigors, the doctor may not have felt justified in withholding quinine any longer, although no positive blood examination has yet been made. The parasites at once disappear from the peripheral blood when quinine is given, so that any examination for them must then be negative.

In England, therefore, it is not at all uncommon for attempts to detect the parasites to fail; and it is a very useful thing to know that there are other features of the blood which afford considerable assistance in arriving at a diagnosis. These other features are:—

*A relative increase in the numbers of the large hyaline lymphocytes in the differential leucocyte count, in association with leucopenia*, or at least no leucocytosis. By leucopenia is meant an actual diminution of the total number of leucocytes per cubic millimetre of blood. The relative increase in large hyaline lymphocytes may be up to 15 or even to 30 per cent., a typical count being, for example—

| | Malarial blood, 4,000 leucocytes per cubic millimetre. | Normal blood for comparison. |
|---|---|---|
| | % | % |
| Small lymphocytes ... ... | 30 | 20 |
| Large hyaline lymphocytes ... | 22 | 8 |
| Polymorphonuclear cells ... | 47 | 60 |
| Coarsely granular eosinophile cells ... ... ... ... ... | 1 | 2 |

A similar high proportion of large lymphocytes in association with leucopenia is at present known to occur in only one other condition, namely, *Kala-azar*, which itself has only recently been proved to be different from malaria. The points which will help to distinguish the two are, first, the history as to what part of the tropics the patient had been in, as kala-azar is mainly acquired in Assam; and secondly, the condition of the spleen, for great and permanent enlargement of this organ is an indication of kala-azar rather than of malaria. People are now beginning to think that the so-called "ague-cake spleen" is not due to real malaria, but to this disease of Assam, kala-azar.

It not infrequently happens that the doctor is glad of any help to aid in determining, in the earlier stages, whether his patient has typhoid fever or not. The kind of case in which this happens is of everyday occurrence, and it is hardly necessary to give examples. Apart from the Widal test, which we will not discuss here, there are two other features of the blood in typhoid fever which may often assist in the diagnosis. The first of these is, that *with typhoid fever there is no leucocytosis*. It is often stated that there is leucopenia, but this is by no means always so. It is, however, an almost absolute rule that the leucocytes are not increased above the average limits of health, unless there is some complication, such as perforation with formation of pus. This fact has often been of service in excluding the diagnosis of typhoid fever; for example, it not infrequently happens that the early symptoms of typhoid fever include a certain amount of abdominal pain, too ill-defined, perhaps, to indicate definite suppuration; and on many occasions a leucocytosis up to 20,000 or more leucocytes per cubic millimetre, has served to exclude typhoid fever and has led to the discovery of an appendicular abscess, or of a pyosalpinx, or of some other similar abdominal suppuration requiring laparotomy.

In addition to the absence of leucocytosis, moreover, there is help to be got from the differential leucocyte count; in typhoid fever the polymorphonuclear cells are relatively diminished, contrary to what is found in suppuration, and at the same time the small *lymphocytes are relatively increased*; this increase of the latter is not as a rule extreme, but it may be very helpful.

It has been stated by one skilled pathologist, that, granted a case of continuous fever which might be typhoid fever, he would be certain of the diagnosis if there were no leucocytosis and at the same time a relative increase in the small lymphocytes in the blood. This is perhaps putting the case rather too strongly; but it indicates how helpful the above examination may be, particularly in excluding typhoid fever when there is a leucocytosis and when the polymorphonuclear cells, and not the small lymphocytes, are relatively increased in the differential leucocyte count.

The leucocytosis of suppuration is well known, but there are a few points in connection with it that are worthy of notice.

In the first place, for suppuration to produce a leucocytosis the pus must be in a confined place. If it is free to escape, it does not as a rule cause leucocytosis. For instance, there may be very extensive suppurative lesions upon the surface of the body, as in impetigo of the skin, without any increase in the number of leucocytes per cubic millimetre of blood; and the same applies to abscesses which have been incised and are draining well, to empyemata that have burst their way into a bronchus, and so on. It is when a collection of pus is under pressure, leading to toxic absorption, that leucocytosis occurs, and this leucocytosis has been particularly studied in connection with appendicitis and appendicular abscesses. The presence or absence of leucocytosis alone, however, is not sufficient to prove the presence or absence of pus under pressure. Many cases of appendicitis in which the leucocytes reach even 25,000 per cubic millimetre of blood, resolve spontaneously; in other cases, especially in chronic cases with a thick wall to the abscess cavity, there may be pus and yet no leucocytosis. It has been found that a rising leucocytosis—that is to say, an increasing number of leucocytes per cubic millimetre of blood on repeated counts—is a strong argument in favour of pus being present; but it is seldom that the busy practitioner can spare the time for repeated counts. It is therefore of considerable moment that *the polymorphonuclear cells are relatively much increased when there is pus,* whatever the total leucocyte count may be. In lobar pneumonia, it is true, there is a similar increase in the polymorphonuclear cells, together with leucocytosis in most cases; so that the blood will not distinguish between lobar pneumonia and a deep-seated abscess. As a rule, however, pneumonia may be determined by the history, symptoms, and physical signs, so that the only misfortune of the similarity of the blood changes in it and in pus formation is that they will not assist one in determining the presence of a pneumonic empyema. The following would be a typical differential leucocyte count in a case of appendicular or other abscess:—

| | From a case of abscess in which the pus was under pressure. Total leucocyte count 18,000 per cubic mm. of blood. % | Normal blood for comparison. Total leucocyte count 8,000 per cubic mm. of blood. % |
|---|---|---|
| Small lymphocytes ... ... | 14 | 30 |
| Large lymphocytes ... ... | 3 | 8 |
| Polymorphonuclear cells ... | 81 | 60 |
| Coarsely granular eosinophile cells ... ... ... ... | 2 | 2 |

It is clear, therefore, that when lobar pneumonia can be excluded, and a deep-seated focus of suppuration is suspected, it is very advisable to make, not only a count of the total number of leucocytes per cubic millimetre of blood, but also a differential leucocyte count in order to determine whether or not the polymorphonuclear cells show a considerable increase relatively to the other white corpuscles in the blood.

